# Gold Standard for Diagnosis of DPN

**DOI:** 10.3389/fendo.2021.719356

**Published:** 2021-10-26

**Authors:** Yongchun Yu

**Affiliations:** Department of Endocrinology, Lhasa People’s Hospital, Lhasa, China

**Keywords:** DPN, QST, neurological scoring system, CCM, HFUS

## Abstract

Diabetic peripheral neuropathy (DPN) is a common complication of diabetes mellitus. It often causes symmetrical paresthesia, loss of sensation, and hyperalgesia. Without early intervention, it might lead to diabetic foot ulceration, gangrene, and subsequent amputation in people with diabetes. DPN is an insidious disease and often underdiagnosed. This paper reviews the current national and international prevalence of DPN, screening methods for early DPN, including quantitative sensory measurement, neurological function scoring system, confocal microscopy, and high-frequency ultrasound, and summarizes the related research progress, clinical application, and development prospects of these methods in recent years.

## Introduction

The global prevalence of diabetes in 2019 is close to 500 million, and is expected to increase by 51% in 2045 (1). According to the Chinese diabetes epidemiological survey, the total prevalence of diabetes and the prevalence of prediabetes are 9.7% (10.6% for men and 8.8% for women) and 15.5%, respectively (2). Diabetic peripheral neuropathy (DPN) is a common chronic complication of diabetes and mainly involves the small nerve fibers. It is diagnosed after the exclusion of other peripheral neuropathies, including autoimmune diseases (Sjogren’s syndrome, lupus, rheumatoid arthritis), infections (HIV, hepatitis B and C), inherited (Charcot-Marie-Tooth), inflammatory (CIDP), tumors, vitamin B12 deficiency, hypothyroidism, alcoholism, and injury or pressure on the nerve. According to the Toronto DPN international consensus for DPN, a definite diagnosis requires at least one symptom and/or at least one sign of neuropathy and abnormality in NCS (3). However, the evaluation of abnormal myelinated nerve fibers in nerve conduction studies, including tactile, proprioceptive, vibration, and motor functions, is the late manifestation of DPN (4). On the contrary, small nerve fiber defects that affect C and A of the pulpless or thin pulp δ nerve fibers involved in thermal response, pain, and autonomic nervous function are thought to occur earlier in DPN (5). Prediabetes, persistent or sporadic pain is usually manifested in the absence of clinically detectable neuropathy, suggesting small nerve fiber defects. Intra-epidermal nerve fiber density from skin biopsy and corneal confocal microscopy (CCM) can detect small nerve fiber damage in prediabetes. Prediabetes is a risk factor for chronic axonal polyneuropathy, which is consistent with the initial involvement of small nerve fibers. This is the main cause of neuropathic pain and incidence rate, and also the starting factor of diabetic foot ulcers (6).

The onset of the disease is insidious and the progress is slow. A small number of patients show neuralgia, while most of the patients may not show symptoms in the early stage and progress to the late stage. Late DPN can cause serious complications, such as diabetic foot ulcers, gangrene, and subsequent amputations, which seriously affect the quality of life of people with diabetes and brings a heavy economic burden.

DPN has a higher incidence in people with diabetes. Studies have shown that almost 50% of people with diabetes will have DPN (7), and in some areas, it can even be as high as 60% to 90% (8). In an epidemiological survey of patients with type 2 diabetes in eight communities in Wuhan and Changshu, China, it was found that the total prevalence of DPN was as high as 71.2% (9). With the improvement of social and economic level, the prevalence of diabetes in children and adolescents is also increasing, and the number of diabetes-related complications such as DPN has also increased (10). Some small cross-sectional studies have shown that the prevalence of DPN in children and adolescents with diabetes ranges from 5% to 62% (11, 12). In the United States, the prevalence of DPN in 1,734 patients with T1D and 258 patients with T2D was 7% and 22%, respectively (7). In Australia, the prevalence of DPN in 1,433 patients with T1D and 68 patients with T2D under 18 years old was 21% and 27% (13), respectively; In Denmark, the prevalence of DPN among 339 T1D adolescents was 62% (14).

The pathogenesis of DPN is complicated and has not yet been fully elucidated. The treatment is limited to intensive blood glucose control and symptomatic treatment. Studies have found that in the early stage of type 1 diabetes, paying attention to DPN and optimizing blood glucose management and control can effectively prevent or delay the occurrence of peripheral neuropathy (14). However, the progress of peripheral neuropathy can only be slowed down by management treatment for type 2 diabetes patients with DPN, but the loss of nerve cells is irreversible (15). Therefore, it is very important to screen and diagnose the peripheral neuropathy of people with diabetes in the early stage and take effective targeted measures in the treatment of DPN.

## Diagnostic Gold Standard of DPN

DPN is defined as “the presence of symptoms and/or signs of peripheral nerve dysfunction in people with diabetes after the exclusion of other causes”. Neuropathy includes the manifestations of the somatic and/or autonomic parts of the peripheral nervous system. It is recommended that there are at least two abnormalities from symptoms, signs, abnormal nerve conduction, quantitative sensory test, or quantitative autonomic nerve test (16). Peripheral neuropathy includes all the conditions leading to the injury of peripheral nervous system and is classified according to the site of nerve injury. Distal symmetrical polyneuropathy (DSP), mononeuropathy, and lumbar/cervical radiculopathy are the most common peripheral neuropathies. The rare sites of peripheral neuropathy include diffuse, length-independent neuropathy, multiple mononeuropathy, multiple radiculopathy, plexopathy, and nerve root exocrine neuropathy. Currently, the diagnosis of DPN is mainly based on characteristic symptoms and signs. Nerve conduction studies (NCS) is one of the gold standard techniques for diagnosing DPN (17). It evaluates the occurrence and development of DPN by detecting the ability of peripheral nerve to transmit electrical signals in patients with DN. NCS has the characteristics of being quantifiable, objective, and sensitive, but it has the following disadvantages: time-consuming, high cost, poor experience, and the need for professional doctors to operate. It is difficult to implement in large sample screening (18). Moreover, NCS is also limited to evaluating large nerve fibers, while small nerve fibers are the first to be affected in DPN patients. These small fiber neuropathies cannot be evaluated by standard electrophysiological tests (19). The application of electrophysiology should be promoted only when the clinical presentation is atypical or the diagnosis is unclear (20). Therefore, it is necessary to apply some simple and effective methods to screen DPN for early intervention and control.

## Early Screening Methods for DPN

### Quantitative Sensory Testing

Quantitative sensory testing (QST) is a technique for evaluating sensory neuropathy. It detects small fiber and large fiber neuropathy through standardized and quantified sensory differences such as stimulation, vibration, and temperature. Compared with NCS, this method is noninvasive and easy to operate. In people with diabetes, sensory dysfunction precedes symptoms such as painful neuropathy and foot ulcers (21). QST, which can reliably measure sensory loss and threshold, provides standardized and quantified stimuli and quantifies response levels. Brown et al. (22) found that cold and hot QST could detect the obvious changes of neurological function in patients with mild to moderate DPN after 12 months, while other indicators (vibration QST, neuropathy score, and monofilament examination) were not sensitive to the changes of neurological function in this population. However, the repeatability of QST has always been a difficult problem in clinical application, and there are significant differences between the treatment courses of the same patient. Relevant studies have shown that according to the severity of neuropathy, the sensitivity of the heat test is variable, with cold damage being 27%–98%, heat damage being 22%–98% (19), vibration testing sensitivity being 58%–84%, and the specificity being 51%–86% (23). A QST-based neural test is used to evaluate vibration perception threshold (VPT), cold perception threshold (CPT), warming perception threshold (WPT), and heat pain perception threshold (HPT). Its sensitivity to vibration test is as high as 84%, and its specificity is as high as 81%. Its sensitivity to heat test is high, and its specificity is medium, and has good repeatability and diagnostic accuracy in evaluating sensory loss (24). In China, Sun Yukai et al. believe that the sensitivity and specificity of VPT for DPN were 85.19% and 88.68%, respectively, and can be used for early screening of DPN lesions (25).

QST also has its limitations. First of all, QST can detect the integrity of the entire sensory nerve axis, which has no value in localization. Secondly, QST is a psychosomatic test, which lacks the objectivity of NCS, and its results will change due to distraction, boredom, mental fatigue, drowsiness, or confusion. When patients consciously or unconsciously prefer abnormal QST results, no psychophysical test can reliably distinguish these patients from patients with organic diseases. Moreover, most of the QST measuring instruments are expensive, the cost is high, the test results are complex, and it is difficult to quantify sensory defects and other factors that hinder the wide application of QST in clinical practice (26).

### Clinical Neurological Function Scoring System

The clinical neurological function scoring system is mainly used in epidemiological surveys for early screening and evaluation of DPN, which helps to grade the severity of the disease and can quantitatively evaluate clinical indicators such as physical examination. Currently, commonly used neurological scoring scales include Michigan neuropathy screening instrument (MNSI), Toronto clinical scoring system (TCSS), neuropathy symptom score (NSS), and neuropathy disability score (NDS).

MNSI was first proposed in 1994 (27), including patient questionnaire and physical examination. The total score is 10 points, and more than 2 points are considered abnormal. It is widely used in clinical practice and large-scale clinical trials to evaluate distal symmetrical peripheral neuropathy, including action to control cardiovascular risk in diabetes (ACCORD) (28) and bypass angioplasty revascularization investigation 2 diabetes (BARI 2D) (29). MDNS includes three parts: toe sensation, distal muscle strength of limbs, and tendon reflex score. The total score is 46 points, and >6 points are considered abnormal. Domestic research has found that the abnormal detection rate of MDNS is 84.5%, the abnormal detection rate of TCSS is 62.0%, the abnormal detection rate of NCV is 76.0%, and the abnormal detection rate of the combination of the three is 91.5%. The sensitivity of MDNS and TCSS are 92.9% and 77.6%, the specificity are 51.6% and 87.1%, the accuracy are 82.9% and 79.8%, the positive predictive value are 85.8% and 95.0%, the negative predictive value are 69.6.% and 55.1%, Youden coefficients are 0.445 and 0.647, Kappa values are 0.488 and 0.539 (*p* < 0.05), and AUC are 0.837 and 0.875, respectively (30). The MNSI composite index has advantages in DPN mid-term screening. TCSS can be used for DPN classification, and its combined application can increase the detection rate of DPN. Some domestic studies believe that with an MNS score of 4 points being the segmentation point (31) and a TCSS score of 5 points being the segmentation point (32), neurological abnormalities can be better diagnosed in China, and the detection rate of DPN has been significantly improved. The limitation of MNS is that when it is used in type 1 people with diabetes with relatively young age, long course of disease, and low prevalence of distal symmetric peripheral neuropathy, the positive predictive value is low and the false positive is high (33).

NSS is scored according to the symptoms, location, and pain relief methods of lower limbs; NDS is scored according to ankle reflex, dorsalis pedis acupuncturing sensation, toe vibration sensation, and temperature sensation. DPN is a mainly axonal disease that gradually develops from the distal end to the proximal end. The nerve damage starts from the most distal end of the limb (34). Early DPN generally occurs in the upper limbs and other parts. In the early stage, sensory neuropathy is more obvious than motor nerve (35). Therefore, NSS and NDS assess lower limb neurosensory function and can be used as a screening tool for early DPN. Liu Wenqu used NSS/NDS to diagnose 679 patients with type 2 diabetes. The results showed that the sensitivity and specificity were 68.0% and 77.2%, respectively, and the positive predictive value, negative predictive value, and Youden index were 86.5%, 53.5%, and 45.2% respectively, suggesting that NSS/NDS has good application value for early screening of DPN patients (36).

### Corneal Confocal Microscope

Corneal confocal microscopy (CCM) is a non-invasive ophthalmic imaging technique for assessing the corneal nerve fiber morphology. With the use of an imaging software, the corneal nerve fiber morphology can be objectively quantified to the corneal nerve fiber density (CNFD), length (CNFL), and branch density (CNBD) of people with diabetes (37).

The application of CCM in diabetes was first proposed about 20 years ago (38), but at that time, due to the limited availability of equipment in general ophthalmology practice, the lack of experts with relevant professional knowledge in corneal scanning, the small field of vision of confocal microscope, the lack of clear consensus on the number of images needed for representative quantitative analysis, and other limitations, it was limited in clinical practice. In addition, severe dry eyes, severe corneal dystrophies, ocular trauma or surgery in the preceding 6 months, and glaucoma can affect the corneal nerve fiber morphology (39).

CCM has experienced three main development stages, namely, series scanning CCM, classified scanning CCM, and laser scanning CCM. At present, it is widely used in clinic. In the field of diabetes, the decrease of corneal nerve density was observed in diabetic animal models. After that, it was found that there were also some changes in the morphology of corneal nerve fibers in diabetic patients, and there was a certain correlation with the degree of peripheral neuropathy. Kallinikos and other scholars believe that this change in nerve fiber morphology is closely related to systemic neuropathy. Therefore, we can observe the changes of corneal nerve fibers through CCM to evaluate DPN.

The literature on the use of IVCM to quantify diabetic neuropathy shows that the density of the corneal basal nerve fibers and the curvature of nerve fibers in diabetic patients are increased. These nerve fiber changes are related to the stage or severity of peripheral neuropathy (39). In addition, ivcm can detect early peripheral neuropathy because the decrease of nerve density precedes the damage of corneal sensitivity. The significant correlation between corneal and cutaneous nerve degeneration in DPN strengthens the evidence that IVCM is a valuable tool for the diagnosis and assessment of DPN (40).

A meta-analysis on the value of CCM in the early diagnosis of DPN confirmed that compared with healthy controls and people with diabetes without DPN, CNFD, CNBD, and CNFL of DPN patients were significantly reduced, and CCM can detect corneal nerve changes in DPN patients (41). In a longitudinal study, 89 patients with type 1 diabetes and 64 healthy subjects were subjected to CCM and clinical and electrophysiological examinations at the same time. Among them, the CCM parameters of DNP subjects were significantly reduced, and the threshold of CNFL ≤ 14.0 mm/mm (2) optimized the sensitivity and specificity of the diagnosis of DNP (sensitivity 85%, specificity 84%) (42).

However, CCM has the advantages of non-invasiveness, simple operation, short surface anesthesia time, and accurate and dynamic observation of corneal nerve changes, among others. In recent years, with the advancement of CCM technology including automatic scanning and analysis and wide-area imaging (43), it has a good application prospect for the evaluation of small nerve fiber damage in DPN, early diagnosis, and treatment effect evaluation.

### High-Frequency Ultrasound

High-frequency ultrasound was first used in the clinical diagnosis of DPN as a supplement to NCS. It measures the size, blood vessels, echo, and mobility of the diseased nerve to show the damage of the nerve tissue, which can effectively improve the diagnostic efficiency of DPN and reduce the missed diagnosis rate and the misdiagnosis rate (44). In patients with DPN, the cross-sectional area (CSA) and longitudinal section of the nerve are increased, and the echogenicity of the nerve, the boundary ambiguity, and the blood flow in the nerve are also significantly increased. The mean CSA in the examined nerves was higher in moderate to severe DPN than the mild DPN (45). High-resolution ultrasound has unique diagnostic advantages for early or subclinical neuropathy. High-frequency ultrasound can detect subclinical involvement of peripheral nerves and abnormalities in patients with normal electrical diagnosis (46).

Studies have shown that the maximum thickness of the median nerve and posterior tibial nerve tract, CSA, and hypoechoic area in people with diabetes are closely related to the degree of neuropathy (*p* < 0.0001) and are significantly greater than the control group (*p* < 0.05) (47), suggesting that high-frequency ultrasound can also be used to grade the severity of DPN. A study shows that the sensitivity and specificity of CSA and vascular proliferation in detecting DPN-induced carpal tunnel syndrome (CTS) are 90.9%, 94.0%, 93.4%, and 90.0%, respectively. The severity of CTS is significantly related to various stages of vascular proliferation, and high-frequency ultrasound can be used to diagnose CTS early and estimate its severity (48). However, some studies have found that NCS is more sensitive than ultrasound when determining the severity of ulnar neuropathy, especially in mild or moderate neuropathy (49).

The ultrasound image of DPN lacks a unified definition, and its results are easily affected by the subjective factors of the examiner. Therefore, it is necessary to establish a complete and unified ultrasound image quantitative scoring system. Some ultrasound scoring systems have been developed. For example, the Bochum ultrasound score (BUS) can effectively distinguish between subacute chronic inflammatory demyelinating polyneuropathy (CIDP) and acute inflammatory demyelinating polyneuropathy (AIDP) (sensitivity 90%, specificity 90.4%) (50). The DCEC scoring system uses the total scores of definition, CSA, echo, and nerve entrapment as quantitative evaluation indicators. The critical value of peripheral nerves in the arms and legs is 14.5, which is a good indicator of the presence or absence of DPN (the area under the curve is 0.85) (sensitivity is 0.81; specificity is 0.80) (51).

In addition, the newly developed ultrasound elastography can evaluate the neuroelasticity of DPN. As a new type of instrument, it can measure the hardness or elasticity of the tissue, reflecting the change of the elasticity of the compressed tissue with the degree of tissue deformation (52). Few studies have previously evaluated the changes in peripheral nerve elasticity in patients with DPN, but some studies have shown that this may be a new breakthrough point. The thickening of the peripheral nerve sheath fibers leads to changes in the elasticity of the tibial nerve in type 2 people with diabetes. The nerve stiffness of people with diabetes without clinical or electrophysiological signs of DPN was significantly higher than that of the control group, with a critical stiffness value of 51.05 kPa, a sensitivity of 90%, and a specificity of 85% (53, 54).

High-frequency ultrasound has the advantages of non-invasiveness and good repeatability, and has broad application prospects in the auxiliary diagnosis and prognosis judgment of DPN. The combination of ultrasound and nerve conduction examination can improve the diagnostic value of DPN and avoid missed diagnosis and misdiagnosis. It is helpful to evaluate the severity and prognosis of DPN with fuzzy boundary, enlarged CSA, and hypoechoic area, but it has certain limitations for the exploration of the deep neuromuscular plexus and lumbosacral plexus. With the popularity of high-resolution ultrasound, especially shear wave elastography, it is expected to replace neuroelectrophysiological examination in the diagnosis of DPN.

In summary, the above methods and technologies have their own advantages and disadvantages and need to be verified and further developed by large samples. They have not been widely used at present and are generally used as a supplement to traditional neuroelectrophysiological examinations. In clinical practice, it is still necessary to establish a unified, accurate, and convenient early screening method and diagnostic grading method for DPN, so as to quantitatively assess the degree of nerve damage, carry out early intervention and management, and improve the quality of life of DPN patients.

## Data Availability Statement

The original contributions presented in the study are included in the article/supplementary material. Further inquiries can be directed to the corresponding author.

## Author Contributions

The author confirms being the sole contributor of this work and has approved it for publication.

## Conflict of Interest

The author declares that the research was conducted in the absence of any commercial or financial relationships that could be construed as a potential conflict of interest.

## Publisher’s Note

All claims expressed in this article are solely those of the authors and do not necessarily represent those of their affiliated organizations, or those of the publisher, the editors and the reviewers. Any product that may be evaluated in this article, or claim that may be made by its manufacturer, is not guaranteed or endorsed by the publisher.
